# Effect of nitric oxide on gene transcription – *S*-nitrosylation of nuclear proteins

**DOI:** 10.3389/fpls.2013.00293

**Published:** 2013-08-01

**Authors:** Alexander Mengel, Mounira Chaki, Azam Shekariesfahlan, Christian Lindermayr

**Affiliations:** Institute of Biochemical Plant Pathology, Helmholtz Zentrum München – German Research Center for Environmental HealthNeuherberg, Germany

**Keywords:** protein *S*-nitrosylation, nitric oxide, post-translational modification, nuclear proteins, redox-modification

## Abstract

Nitric oxide (NO) plays an important role in many different physiological processes in plants. It mainly acts by post-translationally modifying proteins. Modification of cysteine residues termed as *S*-nitrosylation is believed to be the most important mechanism for transduction of bioactivity of NO. The first proteins found to be nitrosylated were mainly of cytoplasmic origin or isolated from mitochondria and peroxisomes. Interestingly, it was shown that redox-sensitive transcription factors are also nitrosylated and that NO influences the redox-dependent nuclear transport of some proteins. This implies that NO plays a role in regulating transcription and/or general nuclear metabolism which is a fascinating new aspect of NO signaling in plants. In this review, we will discuss the impact of *S*-nitrosylation on nuclear plant proteins with a focus on transcriptional regulation, describe the function of this modification and draw also comparisons to the animal system in which *S*-nitrosylation of nuclear proteins is a well characterized concept.

## INTRODUCTION

Nitric oxide (NO) is a small, highly reactive gaseous radical. Although it is cytotoxic in high concentrations, NO plays a key role as a biological messenger in all kingdoms. In plants, it is implicated in various physiological processes like flowering, stomatal closure, germination, root development, gravitropism, and responses to abiotic and biotic stresses (Delledonne et al., 1998; Durner et al., 1998; Garcia-Mata and Lamattina, 2002; Pagnussat et al., 2002; He et al., 2004; Hu et al., 2005; Lombardo et al., 2006; De Michele et al., 2009; Sirova et al., 2011; Tanou et al., 2012).

Due to its instable nature, NO has a very rich chemistry. Besides direct dative binding to metal ions NO can further react with superoxide and molecular oxygen, resulting in the formation of peroxynitrite and dinitrogen trioxide N_2_O_3_ (or higher oxides like NO_2_), respectively. Moreover, adding or removing one electron from the antibonding highest occupied molecular orbital by reducing or oxidizing chemicals yields nitroxyl anion (NO^-^) and nitrosonium cation (NO^+^). Collectively, these species are referred to as reactive nitrogen species (RNS) each having distinct chemical properties leading to numerous reactions with biological molecules like lipids, carbohydrates, nucleic acids, and proteins. Although most of these reactions were assumed to be indicative for nitrosative stress in the past, it has become clear that some of these RNS also function as important redox-signaling molecules in the cell by binding covalently to target proteins (Suzuki et al., 2012; Yun et al., 2012). This as redox-signaling termed mechanism should not be considered as a discrete set of signaling cascades. Rather, the cell should be seen as set of compartments each having distinct redox-sensitive proteins as well as redox buffering capacities. Changes in the redox potential of these compartments could then influence other signaling pathways by modifying redox-sensitive proteins (Foyer and Noctor, 2013).

There are three important NO-dependent modifications: metal nitrosylation, tyrosine nitration, and cysteine *S*-nitrosylation.

In a direct reaction termed metal nitrosylation, NO (Lewis base) binds to the transition metal (Lewis acid) of metalloproteins yielding a metal–nitrosyl complex. One example from mammals is the binding of NO to the heme center of soluble guanylate cyclase which activates this enzyme by inducing conformational changes and this in turn leads to the production of cyclic GMP (Russwurm and Koesling, 2004).

Reactive nitrogen species can modify the activity of proteins by covalently binding to tyrosine and cysteine residues. Tyrosine nitration refers to the addition of a nitro group to susceptible tyrosine residues in ortho position to the hydroxyl group thus leading to 3-nitrotyrosine. The main nitrating species is peroxynitrite which is produced in a diffusion controlled reaction between NO and superoxide (Ferrer-Sueta and Radi, 2009). Tyrosine nitration was originally considered to be indicative for oxidative and nitrosative stress but evidence accumulates that this modification also has a signaling function in plant cells (Cecconi et al., 2009; Gaupels et al., 2011).

*S*-nitrosylation of protein cysteine residues is believed to be the most important mechanism for transduction of bioactivity of NO in plants. The formation of nitrosothiols is still debated. The direct reaction of thiol groups with NO is too slow to occur *in vivo*, instead it is assumed that N_2_O_3_ is the main nitrosylating species in aerobic conditions although the formation of dinitrogen trioxide is controversially discussed (Folkes and Wardman, 2004; Ridnour et al., 2004). Other RNS described to mediate *S*-nitrosothiol formation are nitrosonium and nitroxyl ions (Ridnour et al., 2004). Nitroso groups can also be transferred between thiols in a process termed as transnitrosylation. Transnitrosylation occurs between proteins and between proteins and low molecular weight SNOs (e.g., *S*-nitrosylated glutathione GSNO) in animals; in plants, however, evidence for this mechanism is lacking (Hogg, 2002; Nakamura and Lipton, 2013). Enzymatic denitrosylation is mediated by GSNO reductase (GSNOR) and thioredoxins (Trx), both proteins are crucial for maintaining SNO-homeostasis (Sakamoto et al., 2002; Feechan et al., 2005; Sengupta and Holmgren, 2013).

**FIGURE 1:**
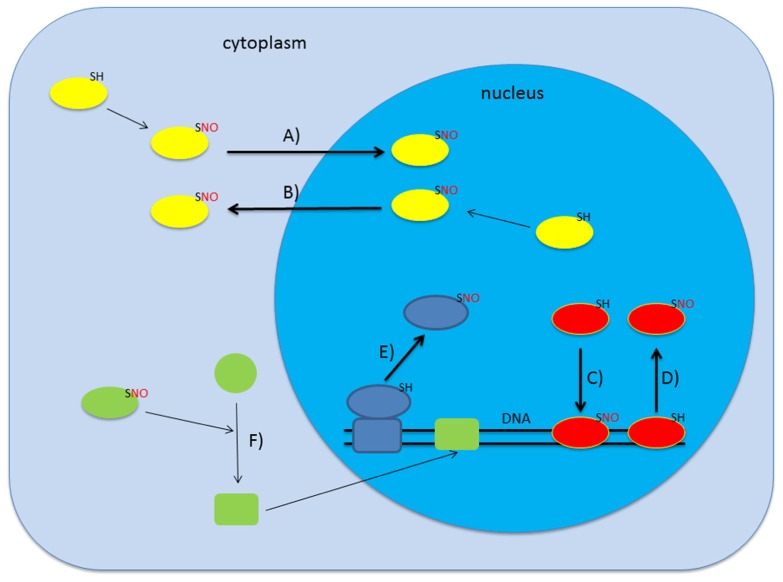
***S*-nitrosylation can affect gene transcription in several ways.** Upon *S*-nitrosylation proteins can change their subcellular localization which may lead to either import in **(A)** or export out **(B)** of the nucleus (Qu et al., 2007; Malik et al., 2010). Alternatively, *S*-nitrosylation is also described to alter DNA-binding activity of certain proteins (**C,D**; Serpa et al., 2007; Lindermayr et al., 2010; Sha and Marshall, 2012). Additionally, SNO-formation can lead to association/dissociation of macromolecular complexes which may result in dissociation from chromatin (**E**; Nott et al., 2008). Various combinations including indirect regulation are also conceivable **(F)**.

Initial proteomic screens for *S*-nitrosylated proteins in *A. thaliana* revealed 53 mainly cytoplasmic proteins but this number increased drastically over the last years (Lindermayr et al., 2005). Up to date several screens targeting the proteomes of different organelles like mitochondria and peroxisomes identified more than 250 candidate proteins to be *S*-nitrosylated involved in a wide range of physiological processes ranging from stress response to metabolism (Kovacs and Lindermayr, 2013; Lounifi et al., 2013). Interestingly, microarray analysis and amplified fragment-length polymorphism (AFLP) transcript profiling of plants treated with gaseous NO and sodium nitroprusside, respectively, showed that NO leads to changes in the transcriptome of *Arabidopsis* (Huang et al., 2002; Polverari et al., 2003). Promoter analysis of the genes co-expressed after NO treatment revealed the accumulation of certain transcription factor binding sites, like octopine synthase gene (ocs) elements and WRKY-sites (Palmieri et al., 2008). This raised the question whether NO affects transcription directly by nitrosylating transcription factors or other transcriptional regulators. In some bacteria, for instance, redox-sensitive cysteine residues of the transcriptional activator OxyR can undergo redox-dependent post-translational modifications like oxidation to sulfinic acid, *S*-glutathionylation, or *S*-nitrosylation. Each of these modifications affects binding affinity and specificity of OxyR to DNA thus resulting in distinct transcriptional responses (Marshall et al., 2000). Besides regulation of DNA-binding, *S*-nitrosylation of nuclear proteins could also affect their subcellular localization or regulate the association with binding partners thereby modulating transcription and/or general nuclear metabolism. In animals, for instance, *S*-nitrosylation of the nuclear export receptor CRM1 (karyopherin chromosomal region maintenance 1) leads to a decrease in the export rate and a subsequent nuclear accumulation of its target protein Nrf2, an antioxidant transcription factor (Wang et al., 2009). The possible modes of action of NO on gene transcription are shown in **Figure 1**.

In this review, we will summarize the current knowledge about *S*-nitrosylated nuclear plant proteins. What is the impact and function of this post-translational modification? Comparisons to the animal system will be drawn in which much more is known about the effect of *S*-nitrosylation on transcription.

## *S*-NITROSYLATED NUCLEAR PROTEINS

### GLYCERALDEHYDE 3-PHOSPHATE DEHYDROGENASE AND CYTOSOLIC ALDOLASE

It is well-known that glyceraldehyde 3-phosphate dehydrogenase (GAPDH) not only plays an important role in glycolysis but also participates in nuclear events like regulation of gene transcription, RNA transport and DNA replication. In animal cells, the link between NO signaling and nuclear action of GAPDH is well established. GAPDH lacks a nuclear localization signal and the homotetramer is too large (150 kDa) to pass passively through nuclear pores. Upon stress GAPDH is specifically nitrosylated at Cys150 by inducible NO-synthase (iNOS) leading to complex formation with seven in absentia homolog 1 (Siah1), an E3 ubiquitin ligase. Siah1 has a very rapid turnover in HEK293 cells but binding to GAPDH markedly increases its stability. The nuclear import signal of Siah1 enables the translocation of the GAPDH/Siah1 complex into the nucleus (Hara et al., 2005). Interestingly, it was shown that nitrosylated GAPDH can transnitrosylate nuclear proteins including deacetylating enzyme sirtuin 1 (SIRT1), histone deacetylase 2 (HDAC2), and DNA-activated protein kinase (DNA-PK) thereby affecting gene transcription (Kornberg et al., 2010). This mechanism can elegantly explain specificity of *S*-nitrosylation in the nucleus in the absence of a nuclear NO-synthase (Stamler and Hess, 2010).

In *Arabidopsis*, both GAPDH isoforms GapC1 and GapC2 were shown to be nitrosylated and glutathionylated on Cys155 and Cys159 (Holtgrefe et al., 2008). These cysteine modifications inhibit GAPDH *in vitro*, but activity could be restored upon addition of dithiothreitol (DTT) demonstrating the reversibility of these modifications. A GFP–GAPDH fusion protein was localized in both the cytosol and nucleus in *A. thaliana* protoplasts indicating partial nuclear localization of GAPDH (Holtgrefe et al., 2008). Moreover, a complex of a GAPDH isoform and NtOSAK (*Nicotiana tabacum* osmotic stress-activated protein kinase) partially localized to the nucleus in BY2 cells after salt stress. Both proteins of this complex seem to be regulated by NO: GAPDH is directly *S*-nitrosylated, whereas the regulation of NtOSAK is rather indirect, involving the NO-dependent phosphorylation of a serine residue in the activation loop of the kinase (Wawer et al., 2010). In addition, cadmium stress induced a strong nuclear accumulation of GapC1 in *Arabidopsis* root tips, which was – in sharp contrast to animal cells – not dependent on *S*-nitrosylation of the catalytic Cys-residue (Vescovi et al., 2013). Interestingly, GAPDH was found to bind to the malate dehydrogenase promoter by using electrophoretic mobility shift assays pointing toward a possible role as transcriptional activator/repressor (Holtgrefe et al., 2008). In conclusion, in contrast to animal cells, the molecular function of *S*-nitrosylation of GAPDH in plants is rather unclear, and further work is needed to decipher the role of GAPDH in NO-mediated signaling.

Aldolases catalyze the reversible condensation of D-glyceraldehyde-3-phosphate and dihydroxyacetone phosphate and are involved in glycolysis, gluconeogenesis, and the Calvin cycle. Higher plants possess different isoforms of aldolases localized to either the cytosol or plastids. It was shown that the enzymatic activity of one isoform of cytosolic aldolase from *A. thaliana* is inhibited by different redox modifications. Cys68 and Cys173 were both glutathionylated, while nitrosylation was only detected at Cys173 (van der Linde et al., 2011). Several studies support the idea that cytosolic aldolase might take over functions in the nucleus. First, this enzyme was found to be localized in the pea leaf nucleus (Anderson et al., 2005). Second, cytosolic aldolase was identified as an interaction partner of the MADS-box transcription factor NMH7 in *Medicago sativa* (Paez-Valencia et al., 2008). Third, a GFP-fusion construct partially localized to the nucleus in *A. thaliana* protoplasts (van der Linde et al., 2011). Fourth, this enzyme was shown to be associated with the NADPH-malate dehydrogenase promoter (Hameister et al., 2007). However, nothing is known about the impact of redox modifications on nuclear activity of cytosolic aldolase.

### MYB TRANSCRIPTION FACTORS

In plants, MYB factors are one of the largest families of transcription factors (Stracke et al., 2001). In the genome of *A. thaliana*, approximately 9% of the estimated number of transcription factors belongs to the MYB family (Riechmann et al., 2000). MYB transcription factors are involved in the regulation of a wide range of physiological processes including metabolic pathways, cell fate and identity, developmental processes and responses to biotic and abiotic stresses (Dubos et al., 2010). They are characterized by a highly conserved DNA-binding domain (MYB DBD). The MYB DBD consists of up to four sequence repeats of about 52 amino acids, each forming three α-helices (Dubos et al., 2010). The third helix of each repeat is the “recognition helix” that makes direct contact with the major groove of DNA (Dubos et al., 2010). The repeated domains increase specificity of DNA-binding and depending on their number, MYB proteins can be divided into different classes. R2–R3 MYB factors constitute an expanded family of MYB proteins in plants that contain a N-terminal DNA-binding domain formed by two adjacent MYB repeats (R2 and R3) and an activation or repression domain usually located in the C-terminus (Dubos et al., 2010). In contrast to the highly conserved MYB domain, the other regions of R2R3-MYB proteins are highly variable which can explain the wide range of regulatory roles of members of this family in plant-specific processes (Wilkins et al., 2009). R2R3-MYB factors contain a highly conserved Cys at position 53 (Cys53) which is also present in MYB proteins from animals and fungi (Serpa et al., 2007). The presence of this surface exposed Cys-residue within the DNA-binding domain raises the question whether DNA-binding activity is regulated by oxidative modifications of this amino acid. Indeed, the DNA-binding of M2D (a fully active DNA-binding domain of AtMYB2) is inhibited by *S*-nitrosylation of Cys53 (Serpa et al., 2007). This mechanism might be conserved throughout different kingdoms as it was demonstrated that NO-donors (SNP and SNOG) severely inhibited DNA-binding of the chicken c-Myb minimal DNA-binding domain R2R3 and that Cys130 (equivalent to Cys53 in plants) is essential for this inhibitory effect (Brendeford et al., 1998).

### NON-EXPRESSOR OF PATHOGENESIS-RELATED GENES 1 AND TGA1

In mammalian immunity, the cofactor inhibitor of kappaB (IκB), which shares structural features with plant non-expressor of pathogenesis-related genes 1 (NPR1; Cao et al., 1997; Ryals et al., 1997), functions to sequester the transcription factor nuclear factor kappaB (NF-κB) in the cytoplasm and prevents it from activating gene expression. In response to pathogen attack, IκB is rapidly phosphorylated and targeted for ubiquitin-mediated proteolysis, allowing NF-κB to localize to the nucleus and activate target genes (Hayden and Ghosh, 2004).

NF-κB itself is a redox-regulated transcription factor in mammals. Within the DNA-binding domain, Cys62 of the p50 subunit is critical for ROS-regulated DNA-binding (Matthews et al., 1992) and is modified by *S*-nitrosylation (Matthews et al., 1996).

The transcription cofactor NPR1, a key regulator of systemic acquired resistance (SAR), is essential for salicylic acid (SA)-mediated signal transduction (Rockel et al., 2002). Recently, it has been shown that NPR1 binds SA and works as a SA receptor (Wu et al., 2012). In unchallenged plants, Cys residues in NPR1 form intermolecular disulfide bonds, driving the formation of NPR1 oligomers (Mou et al., 2003). These NPR1-oligomers are retained in the cytosol. Upon pathogen challenge, the level of SA increases followed by changes in the cellular redox state, resulting in reduction of disulfide bonds in NPR1. Reduction of the NPR1 oligomers releases monomers that translocate to the nucleus where they interact with TGA transcription factors and subsequently activate the expression of pathogenesis-related (PR) genes (Kinkema et al., 2000). Moreover, NPR1 regulates the transcript accumulation of callose synthase genes during defense response (Dong et al., 2008). Interestingly, *S*-nitrosylation of C156 of NPR1 facilitates its oligomerization (Tada et al., 2008). Trx catalyze the monomerization of NPR1 and allow the translocation into the nucleus. Surprisingly, the nuclear translocation of NPR1 is also induced by GSNO (Lindermayr et al., 2010). However, the *S*-nitrosylation-mediated oligomerization is not considered to be an inhibitory effect of NPR1 signaling but rather as a step prior to monomer accumulation.

The TGACG motif binding transcription factors (TGA) belong to the group of basic leucine zipper (bZIP) proteins and the DNA-binding sites for several bZIP factors were enriched in promoter regions of NO-regulated genes (Palmieri et al., 2008). In the nucleus, NPR1 interacts with TGA that binds to *cis*-elements of the *PR1* promoter, promoting *PR1* gene expression and defense (Zhou et al., 2000; Despres, 2003). Redox-dependent interaction with NPR1 is only described for TGA1 and TGA4 which comprise group I and possess four cysteine residues. TGA2, TGA3, TGA5, TGA6, and TGA7 interact with NPR1 independently of the cellular redox status (Zhang et al., 1999; Zhou et al., 2000; Despres, 2003). The Cys residues C260 and C266 of TGA1 form a disulfide bond under oxidizing conditions precluding its interaction with NPR1. These Cys residues are conserved in TGA4, but not in the other TGA isoforms.

Redox regulation of TGA1 and NPR1 has been proposed to involve *S*-nitrosylation (Lindermayr et al., 2010). Both proteins are *S*-nitrosylated *in vitro* after *S*-nitrosoglutathione (GSNO) treatment (Tada et al., 2008; Lindermayr et al., 2010), resulting in enhanced DNA-binding activity of TGA1 toward its cognate target in the presence of NPR1 (Lindermayr et al., 2010). The GSNO-dependent modifications probably result in conformational changes of TGA1 and/or NPR1, which allow a more effective TGA1–NPR1 interaction and enhanced DNA-binding of TGA1 (Lindermayr et al., 2010). The redox status of C172/C287 of TGA1 seems to be important for its DNA-binding activity. Reducing this disulfide bridge and subsequent GSNO-dependent modification of the corresponding cysteine residues positively affect DNA-binding of this transcription factor (Lindermayr et al., 2010).

### HISTONE DEACETYLASES

Acetylation of histone lysine residues is a very important epigenetic regulatory mechanism. Histone acetyltransferases (HATs) catalyze the transfer of acetyl groups from acetyl-coenzyme A on lysine residues of histone tails thereby neutralizing the positive charge of the lysine residue. This reduces the affinity of histones for negatively charged DNA resulting in a loose chromatin structure that is easily accessible for the transcriptional machinery. In contrast, histone deacetylases (HDACs) remove the acetyl group of histone tails and condense the chromatin, thereby resulting in reduced gene expression (Luo et al., 2012). Histones are not the only substrates of HATs and HDACs, acetylation and deacetylation of a wide variety of proteins is catalyzed by these enzymes (Wu et al., 2000). In animals, members of both enzyme groups are known to be regulated by *S*-nitrosylation. Here, we will focus on HDACs because so far there is nothing known about *S*-nitrosylation of HATs in plants*.*

Brain-derived neurotrophic factor (BDNF) and other neurotrophins play a crucial role in the development of the rat and mouse nervous system by influencing the expression of many specific genes that promote differentiation, cell survival, etc. (Nott et al., 2008). Since studies on the effect of NO on chromatin remodeling in neurons showed that NO alters the acetylation state of chromatin associated with the promoter of neurotrophin-regulated genes, one function of NO in the nucleus might be to regulate gene expression by influencing the interaction of transcription factors with chromatin (Nott et al., 2008). Nott et al. (2008) investigated whether NO affects histone acetylation by modifying HDAC activity and found that NO is a key regulator of human histone deacetylase 2 (HDAC2). It was shown that BDNF triggers NO synthesis and also a rapid and sustained *S*-nitrosylation of HDAC2 in neurons. HDAC2 contains three cysteine residues and only double mutation of Cys262 and Cys274 completely abolished its *S*-nitrosylation (Nott et al., 2008). *S*-nitrosylation of HDAC2 did not affect its deacetylase activity, in contrast, it induced its release from chromatin, which lead to an increase of histone acetylation at specific promoter regions and transcription of genes associated with neuronal development including c-fos, egr1, VGF, and nNos (Riccio et al., 2006; Nott et al., 2008). NO-dependent inhibition of HDAC2 function has also been reported in muscle cells (Colussi et al., 2008). Interestingly, *S*-nitrosylation decreases HDAC2 deacetylase activity (Colussi et al., 2008) whereas in neurons HDAC2 enzymatic activity remains unchanged (Nott et al., 2008). This divergence could be due to different *S*-nitrosylated cysteine residue(s) of HDAC2 in muscle cells and neurons (Nott and Riccio, 2009).

 In mammals, class I HDACs are ubiquitously expressed and are localized predominantly in the nucleus. In contrast, class II and IV HDACs are expressed tissue-specific and they are regulated by controlling their subcellular localization (Watson and Riccio, 2009). In unstimulated cells, class II HDACs (e.g., HDAC4/5) are retained in the cytoplasm due to phosphorylation by calcium–calmodulin-dependent kinases (CaMKs) and subsequent association with the cytoplasmic chaperone 14-3-3 (McKinsey et al., 2001). Upon stimulation, dephosphorylation leads to the dissociation of the complex allowing class II HDACs to shuttle into the nucleus. Class II HDACs are indirectly regulated by NO.*S*-nitrosylation of PP2A enforces its binding to HDAC4/14-3-3 leading to dephosphorylation and subsequent nuclear localization of HDAC4 (Illi et al., 2008).

In plants, three families of HDACs can be distinguished based on sequence similarity. The largest family in *Arabidopsis* consists of 12 members – characterized by a highly conserved HDAC domain – and shares homology with yeast RPD3 (reduced potassium dependency protein 3) or HDA1 (histone deacetylase 1). Sirtuins (two members in *Arabidopsis*) are homologous to yeast SIR2 (silent information regulator 2) and have a different catalytic mechanism as they need NADH as a cofactor. The HD2-like family seems to be plant-specific, no homologs have been identified in other organisms so far (Luo et al., 2012). HD2-like proteins play an important role during the hypersensitive response in tobacco: Bourque et al. (2011) showed that NtHD2a/b act as negative regulators of cryptogein induced cell death by using HDAC inhibitors, RNAi, and overexpression approaches. Alignment of *Arabidopsis* RPD3-like HDACs revealed the presence of some highly conserved cysteine residues. Interestingly, Cys262 or Cys274 of human HDAC2 (which were shown to be nitrosylated; Nott et al., 2008) are also preserved in many *Arabidopsis* HDACs (for instance Cys209 and Cys221 of HDA19), making these proteins interesting candidates for further studies. Data from our lab support the idea that histone deacetylases might also be redox regulated in plants (Floryszak-Wieczorek et al., 2012).

## CONCLUSION

*S*-nitrosylation is emerging as one of the most important redox-dependent modifications in plants but only very few detailed studies are available about the impact of this modification on nuclear plant proteins. Important knowledge about *S*-nitrosylation in general in the nucleus is still lacking. Specifically, the presence of NO or nitrosylating species in this compartment has not been proven so far. It is also known that GSH – the main reductant of the cell – accumulates to very high concentrations in the nucleus at certain cell cycle stages, probably to protect the DNA from oxidative damage (Garcia-Gimenez et al., 2013). This raises the question how *S*-nitrosylation in the nucleus is maintained and temporally/spatially controlled. Nevertheless, evidence accumulates that *S*-nitrosylation of nuclear plant proteins (for instance transcription factors) probably participates in regulation of transcription. In animals, several transcription factors are known to be regulated by this post-translational modification: results from studies in neuronal physiology have demonstrated that NO modulates gene expression through the formation of SNO-bonds in multiple transcriptional activators (Nott and Riccio, 2009). For instance, *S*-nitrosylation mediates NO-dependent regulation of various zinc-finger-containing transcription factors, including egr-1 and NFκB. As zinc-finger motifs are very sensitive to *S*-nitrosylation this class of TFs might also be interesting to study in plants. Besides acting on transcription factors, NO also seems to be involved in epigenetic regulation of plant chromatin by modifying key remodeler enzymes like HDACs, which is a new and fascinating aspect of NO-mediated redox signaling in plants. However, important questions are remaining. Work so far has mostly been carried out *in vitro*, the *in vivo* relevance as well as the exact molecular mechanism still needs to be determined leaving much space for future investi - gations.

## Conflict of Interest Statement

The authors declare that the research was conducted in the absence of any commercial or financial relationships that could be construed as a potential conflict of interest.
